# Differential chemokine expression under the control of peripheral blood mononuclear cells issued from Alzheimer’s patients in a human blood brain barrier model

**DOI:** 10.1371/journal.pone.0201232

**Published:** 2018-08-09

**Authors:** Julie Vérité, Guylène Page, Marc Paccalin, Adrien Julian, Thierry Janet

**Affiliations:** 1 EA3808, molecular Targets and Therapeutics of Alzheimer’s disease, University of Poitiers, Poitiers, France; 2 Department of Geriatrics, Poitiers University Hospital, Poitiers, France; 3 Memory Resource and Research Center of Poitiers, Poitiers University Hospital, Poitiers, France; 4 Department of Neurology, Poitiers University Hospital, Poitiers, France; Hungarian Academy of Sciences, HUNGARY

## Abstract

Growing evidence highlights the peripheral blood mononuclear cells (PBMCs) role and the chemokine involvement in the Alzheimer’s disease (AD) physiopathology. However, few data are available about the impact of AD PBMCs in the chemokine signature in a brain with AD phenotype. Therefore, this study analyzed the chemokine levels in a human blood brain barrier model. A human endothelial cell line from the immortalized cerebral microvascular endothelial cell line (hCMEC/D3) and a human glioblastoma U-87 MG cell line, both with no AD phenotype were used while PBMCs came from AD at mild or moderate stage and control patients. PBMCs from moderate AD patients decreased CCL2 and CCL5 levels in endothelial, and also CXCL10 in abluminal compartments and in PBMCs compared to PBMCs from mild AD patients. The CX3CL1 expression increased in endothelial and abluminal compartments with PBMCs from mild AD patients compared to controls. AD PBMCs can convert the chemokine signature towards that found in AD brain, targeting some chemokines as new biomarkers in AD.

## Introduction

In the past, the diagnosis of Alzheimer’s disease (AD) could only be suggested according to clinical symptoms, and a definite diagnosis required autopsy confirmation. However, due to major advances in biomarker and imaging-based research, it is now possible to increase the diagnostic accuracy of AD. Indeed, the combination of the most explored cerebro-spinal fluid (CSF) biomarkers (total tau, hyperphosphorylated tau and the β-amyloid peptide, Aβ42), neuroimaging and neuropsychological tools has been extensively investigated for a number of years and helps to differentiate AD patients from Mild Cognitive Impairment (MCI) and control subjects [1–5]. Although it remains urgent to find biomarkers at the preclinical stage of the disease now revised as a continuum from asymptomatic brain changes to symptomatic stages, many researchers are now moving toward other biomarkers than those of CSF or imaging biomarkers which are invasive and expensive. There are so far no reliable blood biomarkers for neurofibrillary tangle pathology and it is more difficult to establish robust blood biomarkers for plaque pathology [6]. However, AD is also characterized by an inflammatory response [7, 8]. Recently, authors propose that CNS inflammation in AD has many facets. Early inflammation is likely to start as soon as there is a threshold of accumulated Aβ oligomeric peptides well before the presence of amyloid plaques, whereas the late process starts when the first amyloid plaques are established [9]. These both states of inflammation are also in accordance with results in microglia which display an acute activation at early stages of disease with active phagocytosis of Aβ and then a chronic stage at late stages of disease with compromised Aβ clearance [10]. Furthermore, Fan et al. found also two peaks in the AD trajectory, an early protective peak and a later pro-inflammatory peak by using ^11^C-(R)PK11195 and ^11^C-PIB positron emission tomography radiotracers for the translocator protein and amyloid plaques, respectively [11]. The complexity of this inflammatory component also comes from the involvement of the immune system although it is long accepted that the brain is immunologically protected. In AD, the presence of monocytes and lymphocytes has been demonstrated around amyloid plaques [12–15]. The interface between CNS and peripheral immune system is represented by the blood brain barrier (BBB) which involves many cellular and molecular mechanisms to allow communications and this neuroimmune axis formed by the BBB, the immune system and the CNS can interact according to physiological and pathological conditions [16, 17]. In AD, numerous studies indicate a structural alteration of the BBB at late stages, suggesting a broad compensation of this neurovascular unit in an environment marked by an inflammatory reaction, hypometabolism and oxidative stress [18, 19]. The passage of peripheral cells through the BBB is not clear because this passage is not yet highlighted in the early stages of the disease but only at late stages of the disease [12, 13, 15, 20]. Some authors attribute to those cells a more effective role in the Aβ clearance than microglia [21–23]. Besides, other studies revealed that these peripheral blood mononuclear cells (PBMCs) can exacerbate the neuroinflammation [24–26]. Their chemoattraction would better require a thorough analysis at the level of this neuroimmune axis. This analysis is usually performed in isolated biological media and isolated cell lysates while the cellular interaction at the level of the BBB is very strong and can reverse results might be different without taking into account this central and peripheral communication *via* the BBB. To better understand the chemokine role in the pathogenesis of AD, we analyzed their signature at the BBB level where endothelial and abluminal compartments were modeled and did not have AD phenotype. PBMCs were extracted from blood of AD and control patients. Previously, we published a work using the same experimental conditions with PBMCs from AD patients at a moderate stage with Mini Mental State Examination (MMSE) score between 10 and 20. Results showed that PBMCs can modify the chemokine signature at the BBB level and in particular greatly increase the levels of CCL4 and CXCL10 in endothelial and abluminal cells mimicking BBB [27], highlighting the role of immune system in the onset of AD by disturbing the chemokine expression. But this study did not include control patients. In order to better investigate the role of PBMCs at early stage of AD, three groups of patients were included according to neuropsychological examination and MMSE score: moderate AD, mild AD and controls. Results showed that PBMCs from AD patients modified the expression of chemokines in endothelial and abluminal compartments. Indeed, PBMCs from AD patients at a moderate stage can induce a decrease expression of CCL2 and CCL5 in endothelial and abluminal compartments with also a decrease in PBMCs in the BBB model compared to PBMCs issued from patients with mild AD. The chemokine CX3CL1 appears to be an early biomarker because as its expression increased in endothelial and abluminal compartments when PBMCs from mild AD patients were in the BBB model compared to those from controls. Production of CXCL10 was also decreased in PBMCs and abluminal cells in the BBB model with PBMCs from moderate AD patients compared to mild AD patients. This study provides crucial information: PBMCs can modify the cerebral chemokine signature and direct it towards a profile found in brain AD. This may revisit research on the origin of the brain-centered disease. Targeting the immune system would open up new biomarkers for conversion to the disease.

## Material and methods

### Chemical products

Ficoll Histopaque^®^-1077, new born calf serum, phytohaemagglutin (PHA), paraformaldehyde (PFA), sodium fluoride (NaF), phenylmethylsulfonyl fluoride (PMSF), triton X-100, ascorbic acid, human basic Fibroblast Growth Factor (bFGF), hydrocortisone, Fluorescein isothiocyanate-dextran (FD4), Collagen, Type I solution from rat tail, protease and phosphatase inhibitor cocktails and all reagent-grade chemicals for buffers were obtained from Sigma (Saint-Quentin Fallavier, France). RPMI 1640 medium, Dulbecco’s Modified Eagle Medium (DMEM), fetal bovine serum (FBS), 5,000 units of penicillin (base) and 5,000 units of streptomycin (base)/mL mixture (PS), Chemically Defined Lipid Concentrate, trypsine-EDTA (0,05%), 1M HEPES (N-2-hydroxyethylpiperazine-N-2-ethane sulfonic acid) solution, Quant-it^TM^ protein assay kit and ProLong^®^ Gold Antifade Reagent with 4',6-diamidino-2-phenylindole (DAPI) were purchased from FisherScientific (Illkirch, France). Endothelial Basal Medium-2 (EBM-2) from Lonza (Amboise, France). FBS Gold (for EBM-2 medium) were obtained from PAA Laboratories / GE Healthcare Europe GmbH (Velizy-Villacoublay, France). X-MAP^®^ luminex Kit for cytokine assay from Millipore (Molsheim, France). Primary antibodies (goat anti-Iba1 and rabbit anti-NSE) and secondary goat anti-chicken FITC-conjugated antibodies were purchased from Abcam (Paris, France), except for chicken anti-GFAP from Thermofisher (Saint-Herblain, France), donkey anti-goat IgG, donkey anti-rabbit IgG and Bovine Serum Albumine IgG free (BSA) from Jackson ImmunoResearch (Beckman-Coulter, Villepinte/Roissy CDG, France).

### Patients

Thirty-one patients were included in the clinical study “SiTaMA » in the Memory Resource and Research Centre of Poitiers (Poitiers University Hospital, 86021 Poitiers, France). The diagnosis of AD was established according to the National Institute of Neurological and Communicative Disorders and the Stroke-Alzheimer’s Disease and Related Disorders Association (NINCDS-ADRDA) clinical criteria. According to MMSE score, two groups of patients were established: moderate AD with a MMSE score between [10–20] and mild AD with a MMSE score between [21–25]. Patients with no memory complain or cognitive or neurodegenerative disorders were included as controls. Exclusion criteria were: inflammatory disease, chronic or acute infection, neoplasia, treatment with non-steroid anti-inflammatory drug or corticosteroids as these different states could impact the levels of inflammatory mediators.

All patients gave their written informed consent. This study was approved by the institutional review board (“comité de protection des personnes Ouest 3” CPP ouest III, reference 16.01 DC 2016–2017, agreement obtained on March 23^th^ 2016).

### Extraction and culture of peripheral blood monocellular cells

The collection of peripheral blood sample was performed by venipuncture at day 5 after initiation of the Blood Brain Barrier (BBB) model. Blood sample were transferred into 10 mL BD Vacutainer^®^ tube. Then, PBMCs were isolated using a Ficoll Histopaque-1077 density gradient and cultured as previously described [27, 28]. PBMCs were seeded at 500,000 cells/500 μL of complete culture medium (RPMI 1640 medium completed with 10% of newborn calf serum, 1% of PS and 10% of PHA at 20 μg/mL) in 12-well plates for 24 hours, and then transferred in the upper side of insert of the BBB model in direct contact with hCMEC/D3 cells. In most studies, the PBMCs are either stimulated by PHA, lipopolysaccharide (LPS) or β-amyloid peptide (Aβ). We chose the PHA as a mitogen as in our previous studies [27–30] and those of several authors to consider comparisons on chemokine levels [31–33].

### Blood brain barrier model

We decided to use an *in vitro* co-culture model with Transwell inserts to mimick the BBB. Indeed, two cell lines were used: a human endothelial cell line from the immortalized cerebral microvascular endothelial cell line (hCMEC/D3) thanks to Dr Pierre-Olivier Couraud (Cochin Institute in Paris, France) through a Material Transfer Agreement, in order to mimick the BBB. And on the other side we used a human glioblastoma U-87 MG cell line (ATCC^®^ HTB-14^TM^, Manassas in Virginia, USA), displaying markers of astrocytes (glial fibrillary acidic protein, GFAP), microglia (ionized calcium-binding adapter molecule 1, Iba1) and neurons (neuronal specific enolase, NSE) to model the brain parenchyma (see S2 Fig).

U87 cells were cultured in routine in T-75 flask with DMEM medium completed with 10% of FBS and 1% of PS. For the hCMEC/D3 cells, the medium was composed by EBM-2 medium, 5% of FBS gold, 1% of PS, 1.4% μM of hydrocortisone, 5 μg/mL of ascorbic acid, 1% of lipid concentrate, 10 mM of HEPES and 1 ng/mL of bFGF, in a previously coated T-75 flask with collagen type I solution as recommended by Dr Pierre-Olivier Couraud [34]. Respective media were changed every 2 days.

The assembly of the human BBB model followed a very strict chronology as already described in our previous study [35]. Indeed, U87 cells were seeded first (D-1) at a density of 10 000 cells/well in 12-well plates (named as **U87w** in this study), and at the same time on the bottom side of each insert at the density of 3200 cells/insert (called as **U87i**). Inserts were previously pre-coated with collagen type I solution (Transwell Permeable Supports, from Corning). To accomplish this, each transwell was inserted into a 15 mL Falcon tube filled with 5 mL of U87 cells suspension and inverted tube was incubated during 24 hours in incubator to allow cell adherence. The densities of cells, and the timeline to mount the BBB were set to ensure coverage of the surface of the culture plate and insert before each experiment on the BBB model.

The following day (D0), inserts were back in contact with 12-well plates previously seeded with U87 cells, then hCMEC/D3 cells were added on the upper side of each insert, at a density of 50,000 cells/500 μL as recommended by Weksler and collaborators [34]. Media were changed every 2 days.

At day 6, PHA-stimulated PBMCs were added in the chamber of each insert, in direct contact with hCMEC/D3 cells. Finally, the complete BBB model incubated during 48hrs at 37°C in 95% humidified 5% CO_2_ cell culture incubator before collecting supernatants and cell lysates. Furthermore, to study the impact of PBMCs on the BBB model, a BBB model without PBMCs (only U87 and hCMEC/D3 cells) was also prepared in the same experimental conditions and named “BBB model without PBMCs or PB”.

### Cellular lysis

For each patient, the PBMCs were isolated from hCMEC/D3 medium (M1) by centrifugation at 1,500 rpm during 5 min at RT. Supernatants corresponding to M1 and medium of U87 cells (M2) were then directly stored at -80°C until X-MAP^®^ luminex assay. Pellets corresponding to PBMCs were lysed in 150 μL of lysis buffer (50 mM Trizma^®^ base, 50 mM NaCl pH 6.8, extemporaneously supplemented with 1% Triton X100, 1 mM PMSF, 50 mM NaF, 1% protease inhibitor cocktail and 1% phosphatase inhibitor cocktail). The different cell layers (hCMEC/D3, U87i, U87w) were also lysed in 150 μL of lysis buffer. All lysates were then sonicated (output control 2, duty cycle 20%, 5 pulsations) and centrifuged at 15,000 *g* for 15 min at 4°C. The supernatants were saved and analyzed for protein determination using Quant-it protein assay kit with Qubit^®^ material. Samples were frozen at −80°C until further X-MAP^®^ luminex assay.

### Blood brain barrier permeability

Paracellular permeability was assessed by using a molecule tracer (4-kDa Fluorescein Isothiocyanate-Dextran, FD4) as a valuable indicator of barrier integrity. This technique allows us to evaluate the BBB model tightness in our experimental conditions. The flux of FD4 diluted in Hank's Balanced Salt Solution (HBSS) at 50 mg/1,5mL (0.4 g/L KCl, 0.06 g/L KH_2_PO_4_, 8 g/L NaCl, 0.35g/L NaHCO_3_, 0.048 g/L Na_2_HPO_4_, 1 g/L D-Glucose, 0.14 g/L CaCl_2_, 0.1g/L MgCl_2_, 6H_2_O, 0.1g/L MgSO_4_, 7H_2_O) through cellular layers was determined after taking samples (50 μL) from the upper and lower chambers after 0 and 60 min, and each sample was directly transferred into Nunc FluoroNunc/LumiNunc 96-Well plates, then fluorescence was measured by using a Varioskan Flash^®^ microplate reader (Fisher ThermoScientific) with excitation at 485 nm and emission at 515 nm. This BBB permeability assay was performed under two conditions: insert without cells, i.e. only pre-coated insert (control) and BBB model without PBMCs. Generally, permeability values in the order of magnitude of 10^−6^ cm/s are considered good values in scientific literature. In our experimental conditions, the endothelial permeability coefficient was 4.50 ± 0.62 x10^-6^ cm/s (mean ± SEM, n = 10) (S1 Fig).

### Transendothelial electrical resistance measurements

TEER across the luminal side on Transwells was determined using a Millicell ERS-2 (Electrical Resistance System) device (Millipore–[Molsheim] France) with a STX01 electrode. Then, the measured tissue resistance of cells grown on Transwell filter inserts was corrected by deducting the blank resistance evaluated across an empty pre-coated Transwell insert (without cells), and multiplied by the effective surface area (1.12 cm^2^), to give TEER in Ohms×cm^2^ (Ω.cm^2^). In our experimental conditions, results showed that the BBB model yielded TEER values of 37.94 ± 2.33 Ω.cm^2^ (mean ± SEM, n = 31) (S1 Fig), in accordance with values found in other BBB models using the hCMEC/D3 cell line [36].

### Immunofluorescence

U87 cells were grown on coverslips during 48 hrs in 12-well plates in their respective medium. Next these cells were fixed in 4% cold PFA for 15 min, washed twice with sterile Phosphate Buffer Saline (PBS: 154 mM NaCl, 1.543 mM KH_2_PO_4_, 2.7 mM Na_2_HPO_4_,7H_2_O, pH 7.2) for culture cells and permeabilized in PBS (137 mM NaCl, 2.7 mM KCl, 1.7 mM KH_2_PO_4_, 10.14 mM Na_2_HPO_4_, pH 7.4) containing 0.3% of triton-X100 and 5% of BSA. Cells were incubated with specific primary antibodies: either chicken anti-GFAP (1:100), or rabbit anti-NSE (1:50), or goat anti-Iba1 (1:50) at 4°C overnight. After washing, cells were incubated with corresponding secondary antibodies conjugated either with either FITC (anti-chicken IgG and anti-goat IgG), or TRITC or Alexa fluor 488 (anti-rabbit IgG) during 1hr at RT. After final washing, coverslips were mounted on slides with the Prolong Gold antifade reagent with DAPI. The slides were analyzed on a Olympus FluoView™ FV1000 (Olympus, Tokyo, Japan) confocal microscope. Fluorescence signal collection, image construction, and scaling were performed through the control software (Fluoview FV-AS10, Olympus). Multiple fluorescence signals were acquired sequentially to avoid cross-talk between image channels. Fluorophores were excited with 405 nm line of a diode (for DAPI), 488 nm line of an argon laser (for Alexa 488 and FITC), 543 nm line of an HeNe laser (for TRITC). The emitted fluorescence were detected through spectral detection channels between 425–475 nm and 500–530 nm, for blue and green fluorescence respectively and through a 560 nm long pass filter for red fluorescence. The images then were merged as an RGB image (S2 Fig).

### X-MAP^®^ Luminex assay

Human cytokine Luminex custom 5-plex kits (for CCL2, CCL4, CCL5, CXCL10, CX3CL1) were purchased from Millipore. The assay was performed in a 96-well plate and all reagents, standards from a range of concentrations (3.2 to 25,000 pg/mL), quality controls were prepared according to the Millipore instructions. The plates were incubated on a plate shaker at 750 rpm for overnight at 4°C in a darkroom. Assessment was made using luminex-200^®^ instrument and xPONENT® software. 50 beads / assay were collected and median fluorescence intensities (MFIs) were measured. Sensitivity limit was 1.9, 3.0, 1.2, 8.6 and 22.7 pg/mL for CCL2, CCL4, CCL5, CXCL10 and CX3CL1, respectively. MFIs were converted to concentrations (pg/mL) using the equation of standard range of the appropriate chemokine using Milliplex^®^ Analyst Software. Results were expressed as pg/mg protein for cellular lysates and pg/mL in culture media.

### Statistical analysis

Results were analyzed by using GraphPad Prism^®^ software. For all data, we performed the D'Agostino-Pearson normality test that has guided us to the choice of non-parametric tests because the sample size n was too small and that the probability of normality test was greater than 0.05. Comparisons between two groups non matched-pairs were accomplished by using non parametric Mann-Whitney’s test. For more than two groups, non parametric Kruskall-Wallis test followed by Dunn’s test were performed. The level of significance was *P* < 0.05.

## Results

### Patient’s characteristics

Thirty-one patients, 14 men and 17 women, were included in this study and grouped as follow: “control patients” (n = 11, 3 men and 8 women), mean age: 85.6 ± 3.2 years; “mild AD patients” (n = 13, 8 men and 5 women), mean age: 80.2 ± 2.2 years with mean MMSE score: 24.77 ± 0.59); “AD patients at a moderate stage” (n = 7, 3 men and 4 women), mean age: 80.6 ± 2.8 years with mean MMSE score: 19.14 ± 0.40.

### Chemokine expression in BBB models

#### CCL2 levels

The expression of CCL2 in PBMCs from patients with mild AD in the BBB model was increased of 1.6-fold compared to respective PBMCs cultured alone. On the contrary, no differences have been found in the expression of CCL2 in PBMCs from other groups of patients in the BBB models compared to respective isolated PBMCs cells (Table 1). CCL2 expression in D3 cells was strongly increased in the BBB models with PBMCs from controls (26.89-fold) and mild AD patients (29.85-fold) compared to BBB models without PBMCs (Table 2).

**Table 1:** Chemokine levels in PBMCs cultured alone compared to PBMCs in the BBB models.

Chemokine	Group of patients	Isolated cells	PB in BBB
**CCL2**	**Controls**	3193.00 ± 501.70	2695.00 ± 646.90
	**Mild AD**	**2687.00 ± 369.70**	**4290.00 ± 608.00**^**†**^
	**Moderate AD**	2542.00 ± 701.40	2181.00 ± 674.50
**CCL4**	**Controls**	**1618.00 ± 237.80**	44.63 ± 8.42^***^
	**Mild AD**	**894.80 ± 196.50**^**§**^	**46.76 ± 6.27**^**†††**^
	**Moderate AD**	**1082.00 ± 247.00**	**28.79 ± 5.83**^**‡‡‡**^
**CCL5**	**Controls**	**4229.00 ± 1139.00**	242.00 ± 61.29^***^
	**Mild AD**	**2846.00 ± 675.80**	**191.00 ± 34.63**^**†††**^
	**Moderate AD**	**3103.00 ± 724.10**	**108.00 ± 10.73**^**‡‡‡**^
**CX3CL1**	**Controls**	44.17 ± 17.27	30.47 ± 8.02
	**Mild AD**	84.35 ± 22.30	45.39 ± 7.55
	**Moderate AD**	64.89 ± 22.75	29.85 ± 14.03
**CXCL10**	**Controls**	1017.00 ± 146.80	2607.00 ± 525.80
	**Mild AD**	**848.00 ± 201.20**	**4677.00 ± 1578.00**^**†††**^
	**Moderate AD**	858.20 ± 272.10	1234.00 ± 341.70

Expression of CCL2, CCL4, CCL5, CX3CL1 and CXCL10 chemokines in PBMCs in a complete BBB model or cultured alone and prepared from three groups of patients: control patients (n = 11), mild AD patients (n = 13), moderate AD patients (n = 7). Chemokine expression in PBMCs lysates were analyzed by the 5-plex Luminex^®^ xMAP^®^ assay containing a mixture of beads specific for each chemokine as described in Methods. Chemokine levels in lysates are expressed in pg/mg protein.

***P < 0.005 in BBB model with PBMCs from control patients compared to their respective isolated PBMCs

^**†**^*P* < 0.05

^**†††**^*P* < 0.005 in BBB model with PBMCs from mild AD patients compared to their respective isolated PBMCs

^**‡‡‡**^*P* < 0.005 in BBB model with PBMCs from moderate AD patients compared to their respective isolated PBMCs by Kruskal-Wallis test with a Dunns multiple comparison test.

^§^*P*<0.05 in PBMCs from mild AD patients and cultured alone compared to isolated PBMCs from controls by a Mann Whitney’s test.

**Table 2:** Chemokine levels in hCMEC/D3 (D3) in a BBB model with or without PBMCs.

Chemokine	Group of patients	BBB without PB	complete BBB
**CCL2**	**Controls**	**97.13 ± 30.23**	2612.00 ± 451.80**
	**Mild AD**	**97.13 ± 30.23**	**2899.00 ± 311.00**^**†††**^
	**Moderate AD**	97.13 ± 30.23	1692.00 ± 358.60
**CCL4**	**Controls**	**0.54 ± 0.07**	86.23 ± 16.17**
	**Mild AD**	**0.54 ± 0.07**	**72.76 ± 7.93**^**††**^
	**Moderate AD**	0.54 ± 0.07	93.03 ± 43.53
**CCL5**	**Controls**	**14.82 ± 3.70**	553.40 ± 126.40^***^
	**Mild AD**	**14.82 ± 3.70**	**534.6 ± 135.00**^**†††**^
	**Moderate AD**	**14.82 ± 3.70**	**277.90 ± 36.11**^**§§§**^
**CX3CL1**	**Controls**	**3.71 ± 1.12**	170.50 ± 58.54^*^
	**Mild AD**	**3.71 ± 1.12**	**190.00 ± 55.06**^**††**^
	**Moderate AD**	**3.71 ± 1.12**	**155.80 ± 26.67**^**‡‡**^
**CXCL10**	**Controls**	**19.78 ± 7.26**	3780.00 ± 1091.00**
	**Mild AD**	**19.78 ± 7.26**	**4652.00 ± 920.30**^**†††**^
	**Moderate AD**	19.78 ± 7.26	2117.00 ± 614.90

Expression of CCL2, CCL4, CCL5, CX3CL1 and CXCL10 chemokines in lysates of hCMEC/D3 (D3) cells in BBB models with or without PBMCs from three groups of patients: control patients (n = 11), mild AD patients (n = 13), moderate AD patients (n = 7). Chemokine expression in D3 lysates were analyzed by the 5-plex Luminex^®^ xMAP^®^ assay containing a mixture of beads specific for each chemokine as described in Methods. Chemokine levels in D3 lysates are expressed in pg/mg protein.

*P < 0.05

**P < 0.01

***P < 0.005 in BBB model with PBMCs from control patients compared to BBB without PBMCs

^**††**^*P* < 0.01

^**†††**^*P* < 0.005 in BBB model with PBMCs from mild AD patients compared to BBB without PBMCs

^**‡‡**^*P* < 0.01 in BBB model with PBMCs from moderate AD patients compared to BBB without PBMCs by Kruskal-Wallis test with a Dunns multiple comparison test.

^**§§§**^*P*<0.005 in BBB model with PBMCs from moderate AD patients compared to BBB without PBMCs by a Mann Whitney’s test.

In the same way, expression of CCL2 in U87i and U87w cells were highly increased in the BBB models with PBMCs from control patients (by 53.19-fold, and 28.04-fold for U87i and U87w cells respectively), from mild AD patients (by 74.32-fold, and 40.10-fold for U87i and U87w cells respectively) and from moderate AD patients (31.49-fold for U87w cells) *versus* BBB models without PBMCs (Table 3).

**Table 3:** Chemokine levels inU87 (U87i and U87w) cells in a BBB model with or without PBMCs.

Chemokine	Group of patients	U87i in BBB without PB	U87i in complete BBB	U87w in BBB without PB	U87w in complete BBB
**CCL2**	**Controls**	**29.63 ± 6.38**	1576.00 ± 213.40^**^	**76.11 ± 13.68**	**2134.00 ± 345.20**^**‡‡**^
	**Mild AD**	**29.63 ± 6.38**	**2202.00 ± 213.90**^**†††**^	**76.11 ± 13.68**	**3052.00 ± 333.50**^**$ $ $**^
	**Moderate AD**	29.63 ±6.38	1249.00 ± 266.80	**76.11 ± 13.68**	**2397.00 ± 738.50**^**δδ**^
**CCL4**	**Controls**	**0.64 ± 0.08**	19.15 ± 3.38^**^	**0.55 ± 0.06**	**28.78 ± 8.13**^**‡‡**^
	**Mild AD**	**0.64 ±0.08**	**17.70 ± 2.35**^**††**^	**0.55 ± 0.06**	**23.59 ± 2.81**^$ $^
	**Moderate AD**	**0.64 ± 0.08**	**14.92 ± 1.55** ^**γ**^	**0.55 ± 0.06**	**18.78 ± 4.02**^**δδ**^
**CCL5**	**Controls**	**6.61 ± 1.93**	38.84 ± 5.97^**^	**16.48 ± 2.55**	**99.53 ± 15.40**^**‡‡**^
	**Mild AD**	**6.61 ± 1.93**	**67.49 ± 11.62**^**†††**^	**16.48 ± 2.55**	**127.00 ± 18.76**^**$ $ $**^
	**Moderate AD**	6.61 ± 1.93	21.22 ± 4.80	16.48 ± 2.55	65.32 ± 15.03
**CX3CL1**	**Controls**	13.91 ± 3.24	80.10 ± 33.55	**9.97 ± 2.27**	**119.00 ± 23.14**^**‡‡**^
	**Mild AD**	**13.91 ± 3.24**	**93.04 ± 18.88**^**††**^	**9.97 ± 2.27**	**118.80 ± 18.56**^**$ $ $**^
	**Moderate AD**	**13.91 ± 3.24**	**143.80 ± 35.83**^**γ γ γ**^	**9.97 ± 2.27**	**107.80 ± 22.63**^**δδ**^
**CXCL10**	**Controls**	**33.87 ± 15.29**	2385.00 ± 657.50^*^	**29.66 ± 10.71**	**2522.00 ± 556.90**^**‡‡**^
	**Mild AD**	**33.87 ± 15.29**	**2699.00 ± 432.60**^**††**^	**29.66 ±10.71**	**2786.00 ± 487.20**^**$ $ $**^
	**Moderate AD**	33.87 ± 15.29	972.60 ± 235.80	29.66 ± 10.71	1454.00 ± 455.00

Expression of CCL2, CCL4, CCL5, CX3CL1 and CXCL10 chemokines in lysates of U87 cells in BBB models with or without PBMCs from three groups of patients: control patients (n = 11), mild AD patients (n = 13), moderate AD patients (n = 7). **U87i** are U87 cells seeded on the external side of the inserts, while **U87w** are U87 cells seeded on bottom wells. Chemokine expression in U87 lysates were analyzed by the 5-plex Luminex^®^ xMAP^®^ assay containing a mixture of beads specific for each chemokine as described in Methods. Chemokine levels in U87 lysates are expressed in pg/mg protein.

*P < 0.05

**P < 0.01 in BBB model with PBMCs from control patients compared to U87i in BBB models without PBMCs

^**††**^*P* < 0.01

^**†††**^*P* < 0.005 in BBB model with PBMCs from mild AD patients compared to U87i in BBB models without PBMCs

^**γ γ γ**^*P* < 0.005 in BBB model with PBMCs from moderate AD patients compared to U87i in BBB models without PBMCs by Kruskal-Wallis test with a Dunns multiple comparison test.

^**‡‡**^*P* < 0.01 in BBB model with PBMCs from control patients compared to U87w in BBB models without PBMCs

^**$ $**^*P* < 0.01

^**$ $ $**^*P* < 0.005 in BBB model with PBMCs from mild AD patients compared to U87w in BBB models without PBMCs

^**δδ**^*P* < 0.01 BBB model with PBMCs from moderate AD patients compared to U87w in BBB models without PBMCs by Kruskal-Wallis test with a Dunns multiple comparison test.

**Fig 1:**
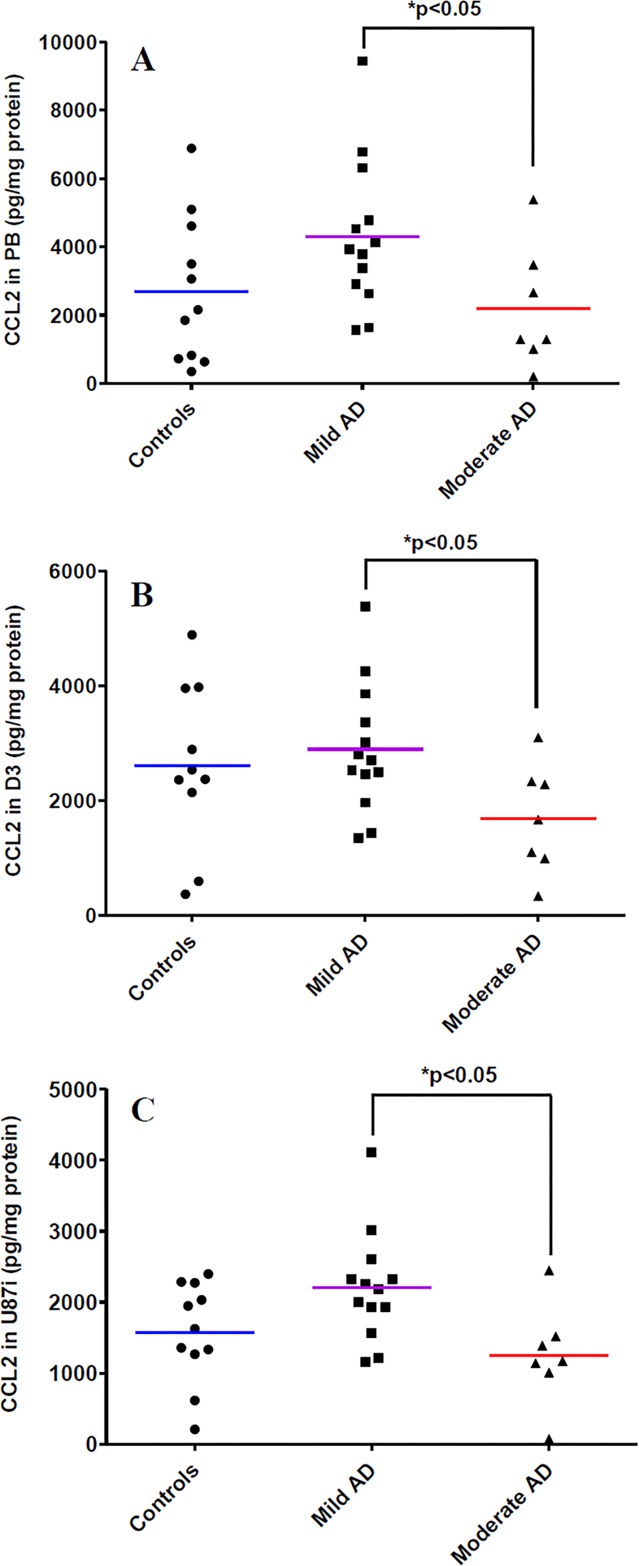
CCL2 levels in experimental human BBB models. CCL2 expression in lysates of PBMCs (PB) **(A)**, in hCMEC/D3 cells (D3) **(B)** and in U87 cells seeded on the external side of the insert (U87i) **(C)**. PBMCs were extracted from three groups of patients: control patients (n = 11), mild AD patients (n = 13), and moderate AD patients (n = 7). Chemokines were analyzed by the 5-plex Luminex^®^ xMAP^®^ assay containing a mixture of beads specific for each chemokine as described in Methods. Chemokine levels in lysates are expressed in pg/mg protein. The mean is represented by a colored line following PBMCs origins (blue, purple, red for control, mild AD and moderate AD patients, respectively). *P < 0.05 in BBB model with PBMCs from moderate AD patients compared to BBB model with PBMCs from mild AD patients by a Mann-Whitney’s test.

However, in our experimental conditions, CCL2 expression decreased in PBMCs, D3 and AT lysates in the BBB models with PBMCs from moderate AD patients, *versus* that in the BBB models with PBMCs from mild AD patients by 1.97-fold, 1.71-fold, and 1.76-fold respectively (Fig 1). No differences have been observed in the expression of CCL2 in M1 and M2 culture media between each group of patients (data not shown).

#### CCL4 levels

As shown (Table 1), a significant decrease in the expression of CCL4 was found when PBMCs were in contact with the BBB models compared to PBMCs cultured alone. Indeed, a respective decrease by 36.25-fold, 19.14-fold and 37.58-fold for control, mild AD and moderate AD patients was observed in PBMCs in the BBB model. On the contrary, expression of CCL4 in D3 lysates in the BBB model was increased by 159.70-fold, 134.74-fold and 172.27-fold with control, mild AD and moderate AD patients respectively, compared to BBB models without PBMCs (Table 2). In the same way, expression of CCL4 in U87i and U87w cells were strongly increased in the BBB models with PBMCs from control patients (by 29.92-fold, and 52.33-fold for U87i and U87w cells respectively), and from mild AD patients (by 27.65-fold, and 42.89-fold for U87i and U87w cells respectively) and from moderate patients (by 23.31-fold and 34.20 fold for U87i and U87w cells respectively) *versus* BBB models without PBMCs (Table 3). The CCL4 expression in PBMCs cultured alone obtained from mild AD patients was lower by 1-81-fold compared to PBMCs from control patients (Table 2). No differences have been observed in culture media (data not shown), or in the other lysates (Tables 2 and 3).

#### CCL5 levels

**Fig 2:**
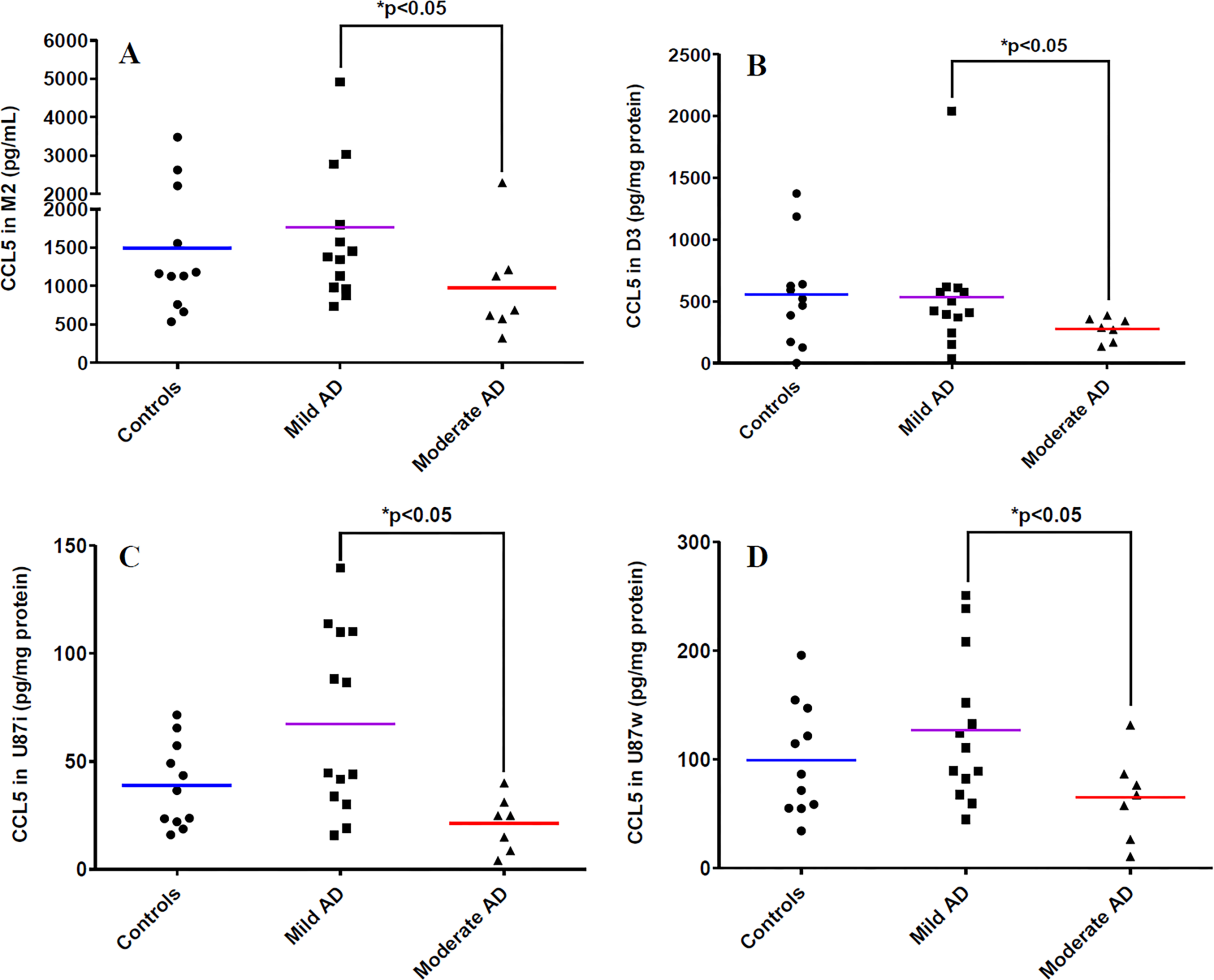
CCL5 levels in experimental human BBB models. CCL5 expression in the abluminal culture media M2 **(A)**, in hCMEC/D3 cells (D3) **(B)**, in U87 cells seeded on the external side of the insert (U87i) **(C)** and in U87 cells seeded on bottom wells (U87w) **(D)**. PBMCs were extracted from three groups of patients: control patients (n = 11), mild AD patients (n = 13), and moderate AD patients (n = 7). Chemokines were analyzed by the 5-plex Luminex^®^ xMAP^®^ assay containing a mixture of beads specific for each chemokine as described in Methods. Chemokine levels in lysates are expressed in pg/mg protein, and in pg/mL for culture media. The mean is represented by a colored line following PBMCs origins (blue, purple, red for control, mild AD, and moderate AD patients, respectively). *P < 0.05 in BBB model with PBMCs from moderate AD patients compared to BBB model with PBMCs from mild AD patients by a Mann-Whitney’s test.

Results showed a decrease in the expression of CCL5 in PBMCs in the BBB models by 17.48-fold for control patients, 14-90-fold for mild AD patients and 28.73-fold for moderate AD patients *versus* PBMCs cultured alone (Table 1). On the contrary, CCL5 levels in D3 lysates were increased in the BBB models with PBMCs from control patients (a 37.34-fold increase), from mild AD patients (a 36.07-fold increase) and from moderate AD patients (by 18.75-fold increase) *versus* D3 lysates in BBB model without PBMCs (Table 2). The increased expression of CCL5 was also observed in U87i and U87w lysates in control patients (by 5.88-fold and 6.04-fold respectively) and in mild AD patients (by 10.21-fold and 7.70-fold respectively) *versus* respective U87 lysates in BBB models without PBMCs (Table 3). In addition, a decreased expression of CCL5 was found in D3 lysates (by 1.92-fold), in U87i lysates (by 3.18-fold) and in U87w lysates (by 1.94-fold) in the BBB model with PBMCs from moderate AD patients *versus* mild AD patients. This decreased expression of CCL5 in moderate AD patients was observed as well in M2 culture media (1.81-fold), compared to mild AD patients (Fig 2).

#### CX3CL1 levels

**Fig 3:**
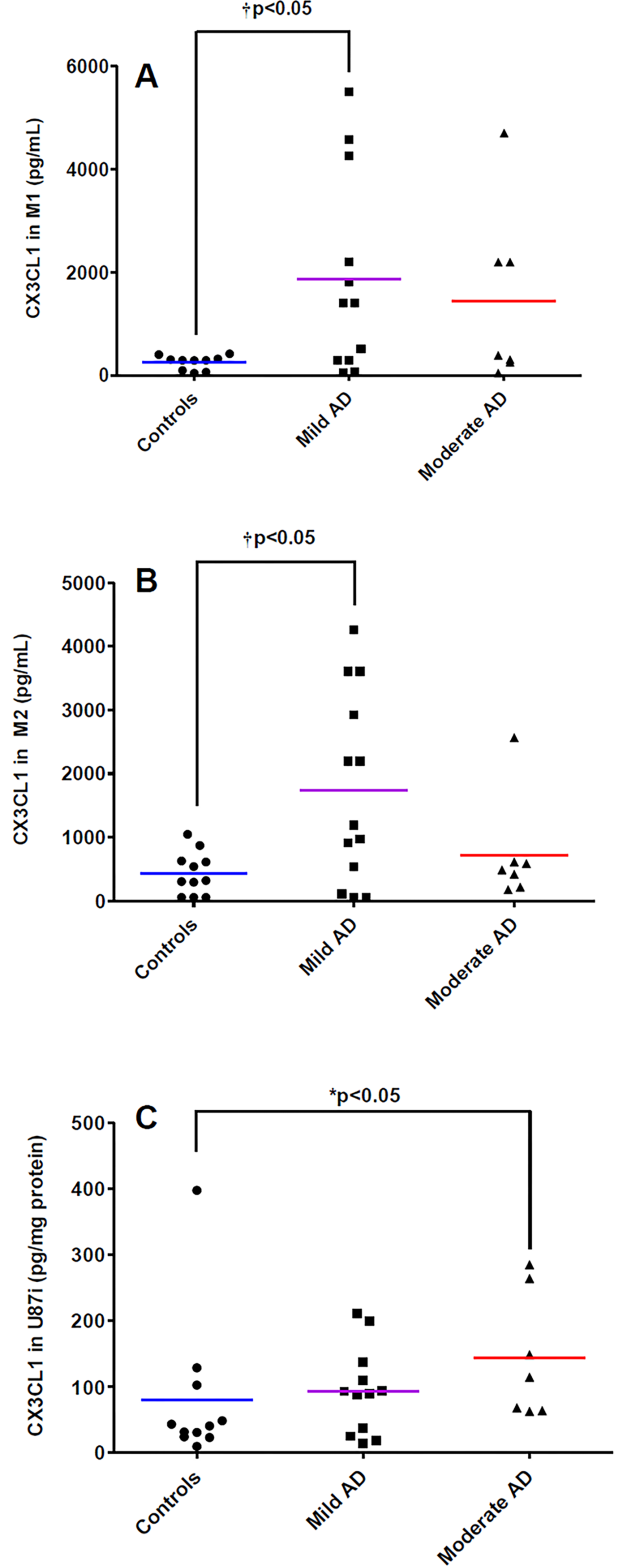
CX3CL1 levels in experimental human BBB models. CX3CL1 expression in the luminal culture media M1 **(A),** in the abluminal culture media M2 (M2) **(B)** in U87 cells seeded on the external side of the insert (U87i) **(C)**. PBMCs were extracted from three groups of patients: control patients (n = 11), mild AD patients (n = 13), and moderate AD patients (n = 7). Chemokines were analyzed by the 5-plex Luminex^®^ xMAP^®^ assay containing a mixture of beads specific for each chemokine as described in Methods. Chemokine levels in lysates are expressed in pg/mg protein, and in pg/mL for culture media. The mean is represented by a colored line following PBMCs origins (blue, purple, red for control, mild AD, and moderate AD patients, respectively). *P < 0.05 in BBB model with PBMCs from moderate AD patients compared to BBB model with PBMCs from control patients and ^†^*P* < 0.05 in BBB model with PBMCs from mild AD patients compared to BBB model with PBMCs from control patients by a Mann-Whitney’s test.

No differences have been observed in the expression of CX3CL1 in PBMCs in the BBB models compared to PBMCs cultured alone (Table 1). However, CX3CL1 levels in D3 lysates were increased when PBMCs from control patients (by 45.95-fold), mild AD patients (by 51.21-fold) and from moderate AD patients (by 42.00-fold) were in contact with the BBB model, compared to D3 in BBB without PBMCs (Table 2). In addition, as shown in Table 3, CX3CL1 levels were also increased in U87i lysates in BBB models with PBMCs from mild AD patients (6.69-fold) and moderate AD patients (10.34-fold) compared to U87i lysates in BBB models without PBMCs. In the same way, CX3CL1 expression was higher in U87w lysates in BBB models with PBMCs from controls patients (11.93-fold), mild AD patients (11.92-fold) and moderate AD patients (10.81-fold) (Table 3). In our experimental conditions, an increased expression of CX3CL1 (1.80-fold) was found in U87i lysates in the BBB model with PBMCs from moderate AD patients *versus* control patients (Fig 3). Furthermore, CX3CL1 levels in M1 and M2 culture media were increased by 7.43-fold and 4.01-fold respectively in the BBB model with PBMCs from mild AD patients *versus* control patients (Fig 3).

#### CXCL10 levels

**Fig 4:**
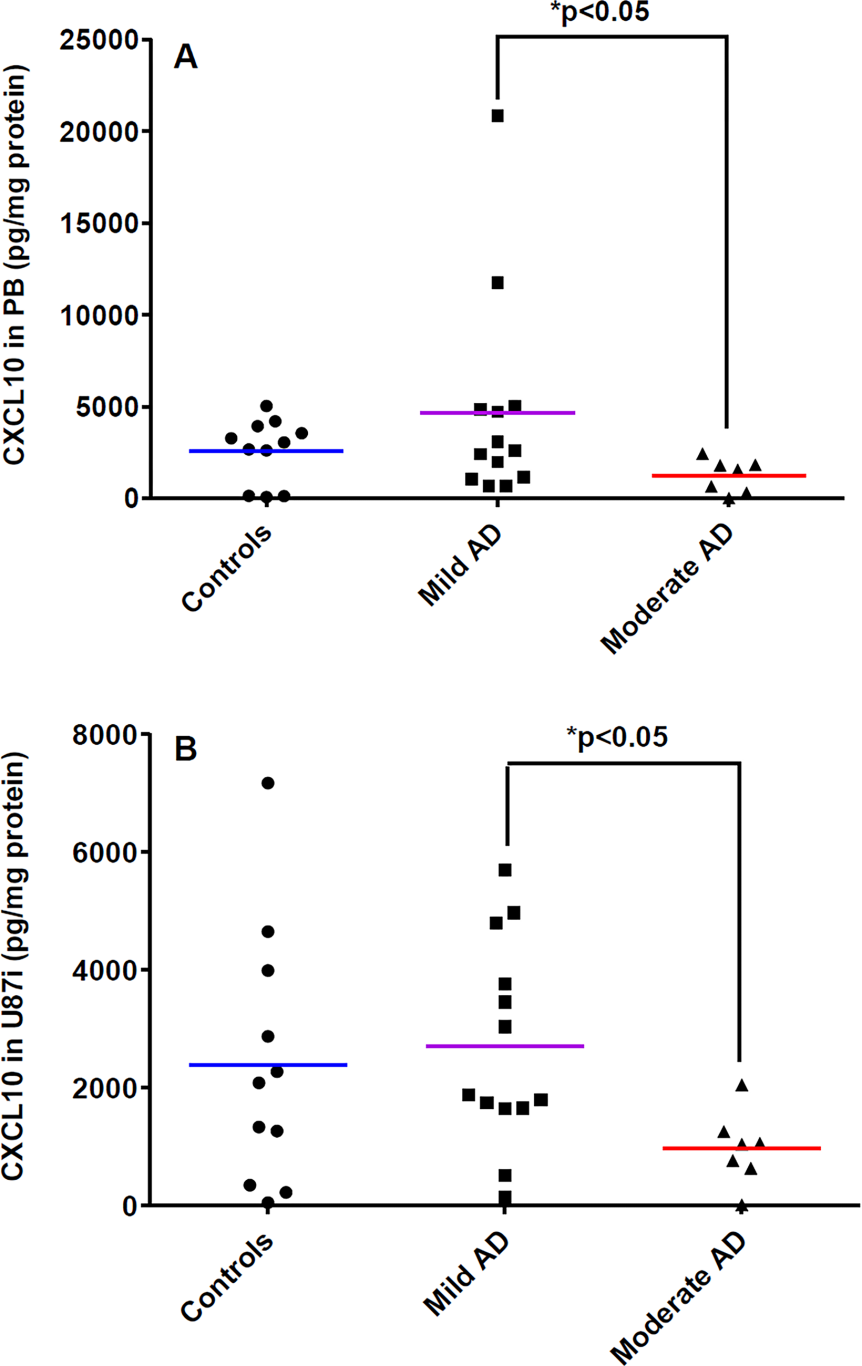
CXCL10 levels in experimental human BBB models. CXCL10 expression in lysates of PBMCs cells (PB) **(A)** and in lysates of U87 cells seeded on the external side of the insert (U87i) **(B)**. PBMCs were extracted from three groups of patients: control patients (n = 11), mild AD patients (n = 13), and moderate AD patients (n = 7). Chemokines were analyzed by the 5-plex Luminex^®^ xMAP^®^ assay containing a mixture of beads specific for each chemokine as described in Methods. Chemokine levels in lysates are expressed in pg/mg protein. The mean is represented by a colored line following PBMCs origins (blue, purple, red for control, mild AD and moderate AD patients, respectively). *P < 0.05 in BBB model with PBMCs from moderate AD patients compared to BBB model with PBMCs from mild AD patients by a Mann-Whitney’s test.

As shown in Table 1, an increase by 5.51-fold of CXCL10 expression was only found in PBMCs lysates from mild AD patients in the BBB models when compared to PBMCs cultured alone. This increased expression of CXCL10 was also found in D3 lysates in the BBB model with PBMCs from control patients (by 191.10-fold) and mild AD patients (by 235.1-fold) compared to D3 lysates in BBB models without PBMCs (Table 2). In the same way, CXCL10 expression in U87i and U87w lysates was increased in BBB model with PBMCs from control patients (by 70.41-fold and by 85.03-fold respectively) and from mild AD patients (by 79.69-fold and by 93.93-fold respectively) *versus* U87i and U87w lysates in BBB models without PBMCs (Table 3). Results also showed a decrease in the CXCL10 levels in PBMCs (3.79-fold) and U87i (2.78-fold) lysates in the BBB models with PBMCs from moderate AD patients *versus* BBB models with PBMCs from mild AD patients (Fig 4).

## Discussion

It is now widely accepted that inflammation plays an important role in the pathogenesis of AD [37]. Besides, a prominent neuroinflammation occurring in the brain parenchyma during AD, a peripheral inflammation including the active involvement of peripheral immune cells such as T lymphocytes or monocytes is observed [24]. Those observations imply that peripheral immune cells are able to cross BBB under specific conditions [12, 38]. To go further, chemokines which are chemotactic cytokines play a critical role in many biological processes including the recruitment of peripheral cells to their final target such as the site of a lesion [39]. Interestingly, some authors showed a direct impact of chemokines in the pathogenesis of AD [40, 41]. Most studies focus on their production at a central level in the parenchyma brain by measuring their expression either in the CSF [42, 43], or at a cellular level in microglia or astrocytes [44], which are key actors in neuroinflammation processes during AD [8, 10, 45, 46].

Peripherally, most available data come from measurements of chemokine levels in biological media such as plasma or serum of patients [47], or in cellular actors like PBMCs [48]. Studies analysing the chemotactic crosstalk occurring at the BBB between peripheral cells, actors of the BBB and the parenchyma brain during AD are lacking. We conducted a previous study in order to analyse the production of five chemokines known to be implicated in the pathogenesis of AD in a human BBB model, and also to assess whether PBMCs from AD patients could impact chemokine production [35]. This work showed that BBB models are valuable tools to explore chemokine production and that PBMCs from AD patients at a moderate stage could influence CCL4 and CXCL10 production in this human BBB model. However, data concerning the potential impact of PBMCs from control and mild AD patients were lacking. To overcome this issue, we decided to assess the chemokine production (CCL2, CCL4, CCL5, CX3CL1 and CXCL10) in a human BBB model with PBMCs obtained from three groups of patients as follow: controls, mild AD and moderated AD patients. In this particular setting, we wanted to evaluate if chemokine production could be different according to the severity of AD, and potentially detect useful biomarkers to help the following of AD progression or to propose therapeutic targets. Since we had some issues with the H4 cell line used in our previous work, we decided to use the U87 cell line to mimic the brain parenchyma along with the hCMEC/D3 endothelial cell line to form our human BBB model. The chemokine production in a human BBB model without PBMCs from patients was also evaluated.

This human endothelial cell line has a different transcriptional profiling compared to freshly isolated mouse brain microvascular endothelial cells (BECs), in particular a great reduction of expression of the claudin-5, occludin and JAM2, likely explaining its low TEER. Furthermore, the hCMEC/D3 endothelial cell line expresses low levels of endothelial transporters such as Glut1 and P-gp and of cell surface receptors such as LRP1, RAGE and the insulin receptor [49]. In our study, we analyzed the chemokine profile in the human BBB model including this human cell line. Chemokines are a group of small (8–14 kDa) structurally related molecules released by a variety of cell types. It is known that some chemokines go through BBB by a transcellular transport (interaction with Duffy antigen receptor [50], glycosaminoglycans [51] such as CCL2, CXCL8 and CXCL12. In addition, these small soluble chemokines can cross the pores of the insert which are 0.4 μm in diameter.

Results showed that as reported in our previous work [35], that PBMCs can impact chemokine production in our BBB model, and in return, actors of the BBB model can influence chemokine expression in PBMCs. Indeed, CCL2 and CXCL10 levels were increased in PBMCs when they were in contact with the BBB model compared to PBMCs cultured alone and CCL4 and CCL5 were decreased in the BBB model in comparison to isolated cells. Interestingly, this effect was mostly observed when PBMCs were obtained from controls patients or with AD patients at a mild stage. In addition, when PBMCs were added in the BBB model, an increase production of chemokines was measured both in D3 and U87 cell lysates, compared to BBB model without PBMCs, also mostly with PBMCs obtained from controls and mild AD patients.

### CCL2

CC chemokine ligand 2 (CCL2) also known as “Monocyte Chemoattractant Proteins 1” (MCP-1) is a key member of the CC-chemokine sub-family. CCL2 is expressed by a vast number of cellular actors such as smooth muscular cells, monocytes, astrocytes, microglial cells, and neurons. CCL2 through its receptor CCR2 exerts several biological effects, and is known to be implicated in neurodegenerative processes such as AD [52, 53]. In the present study, we showed that CCL2 expression in PBMCs, D3 and U87i lysates was decreased in the BBB model with PBMCs obtained from patients with moderate AD compared to patients with mild AD. These results are consistent with the literature where authors found that serum CCL2 expression is diminished in severe AD, while it is elevated in MCI and mild AD patients [54]. In addition, CCL2 intrathecal levels increase during AD and stay elevated for patients with severe AD [42, 43]. Concerning the beneficial or deleterious role of CCL2 in the pathogenesis in AD, results are more controversial. It seems like there is a need to strike the right balance between CCL2 and CCR2 expression. Indeed, some authors demonstrated that over-expression of CCL2 in a murine APP/CCL2 model leads to cognitive dysfunctions with an accelerated β-amyloidosis, and gliosis [55]. The same pattern has been observed with mouse models deficient for CCR2 [56]. On the contrary, while other authors studied the effect of CCL2 in a murine APP/CCL2 KO model, they found that the lack of CCL2 production caused an alteration in cognitive functions, associated with a stronger and accelerated β-amyloidosis, and microglial dysfunction [57]. CCL2 seems to be a double-edged sword in the pathogenesis in AD, therefore it makes it rather difficult to consider it as a therapeutic target. On the other hand, CCL2 could be a good candidate as a biomarker to follow-up the conversion of mild AD patients to moderate AD patients, and our BBB model could be a useful tool to detect the decrease of CCL2 over the progression of AD.

### CCL4

CC chemokine ligand 4 (CCL4), also named “Macrophage Inflammatory Protein 1-β”(MIP1-β) is a member of the CC-chemokine sub-family as well. This chemotactic cytokine can both act as a chemoattractant or can induce the production of pro-inflammatory molecules such as IL-1 or IL-6 [58]. In our experimental conditions, the production of CCL4 is decreased in PBMCs cultured alone and obtained from patients with mild AD compared to PBMCs from control patients. Little is known in the literature about the production and the role of CCL4 in the pathogenesis of AD. Our laboratory previously showed that PBMCs from patients with moderate AD were able to induce the production of CCL4 especially in H4 and hCMEC/D3 cells in a human BBB model [35]. Here, we found also that PBMCs induced CCL4 production in hCMEC/D3 and U87 cells. Other authors demonstrated that primary monocyte-derived macrophages and primary adult astrocytes under an amyloid- Aβ treatment were able to produce CCL4 [59]. The work of Zhu *et al* [60] showed that there is an over-expression of CCL4 mRNA in the brain of mouse APP/PS1 models that was directly correlated with the age-related progression of the amyloid- Aβ pathology in this model. In the same way, Martin *et al* [61] demonstrated that CCL4 production is increased in the brain parenchyma of two mouse models of AD (TgAPP/PS1 and TgAPP/PS1dE9). Results showed that PBMCs are able to influence CCL4 levels at the BBB but CCL4 levels can not discriminate control, mild AD and moderate AD patients excepted in PBMCs cultured alone. In mouse models, brain CCL4 levels also increased in wild-type mice with aging [61]. More studies are needed to have a better understanding of the role of CCL4 in the pathogenesis of AD.

### CCL5

CCL5 or Regulated on Activation, Normal T Expressed and Secreted (RANTES) and its receptor CCR5 are found in a wide number of cell types such as endothelial cells, glial cells or immune cells [40]. CCL5 seems to be implicated in several biological processes such as the neuromodulation of glutamate or can act as a chemoattractant for T cells, monocytes, dendritic cells or Natural Killer cells [58]. In the present study, CCL5 expression is decreased in hCMEC/D3 cells, U87 cells and in the abluminal culture media M2 within the human BBB model with PBMCs from moderate AD patients compared to the human BBB with PBMCs from mild AD patients. Interestingly, other authors showed that CCL5 and its receptor seem to be up-regulated in PBMCs from patients with AD compared to control patients [48, 62]. It is important to note that in this study both patients with mild AD and moderate AD were mixed in the same group as AD patients [48]. In the same way, Iarlori *et al* [31] found higher levels of CCL5 in PBMCs from AD patients compared to controls, but the mean ± SD of MMSE score of AD patients was 21 ± 3, most of the patients included were mild AD patients. Here we found that CCL5 expression could evolve and decrease during the progression of AD, especially in moderate AD patients. Concerning its role, CCL5 may play a role in neuroprotection. Indeed, CCL5 improved neuronal survival *in vitro* against sodium nitroprusside toxicity, or thrombine [63]. Moreover, in this study CCL5 expression was stimulated under oxidative stress. Bruno *et al* [64] showed that neuronal cultures were protected against Aβ treatment with CCL5. Regarding these results, CCL5 could be a good therapeutic target, and its supplementation especially in late stages of AD could be potentially beneficial.

### CX3CL1

CX3CL1 also known as Fractalkine is a member of the sub-family CX3C. CX3CL1 is constitutively expressed in neurons localised in the hippocampus and the cerebral cortex [65]. Microglia does not express CX3CL1, but its receptor C-X3-C Motif Chemokine Receptor 1 (CX3CR1). CX3CL1 exists in two forms: one membrane bound-form and one secreted form that acts like an adhesion molecule and allows the interaction with microglial cells. CX3CL1 and CX3CR1 play an important role in the communication between neurons and microglia [66]. Concerning its implication in the pathogenesis of AD, CX3CL1/CX3CR1 may play at the same time a positive and a deleterious role. Indeed some authors showed that CX3CL1/CX3CR1 had a neuroprotective role in a rat Parkinson’s disease model [67] and prevented oxidative stress in murine glial and neuronal cells *in vitro* [68]. While other authors found that it could had a deleterious effect and contribute to the pathogenesis of AD [66, 69]. In our experimental conditions, we found that CX3CL1 expression was increased in U87i cells in the BBB model with PBMCs obtained from patients with moderate AD compared to control patients. Whereas an increase in both M1 and M2 culture media of CX3CL1 production was observed in the BBB model with PBMCs obtained from patients with mild AD compared to control patients. Those results are consistent with the literature. Indeed, Kim *et al* [47] showed that the expression of soluble CX3CL1 was higher in the plasma of patients with mild to moderate AD compared to patients with severe AD. Interestingly, they found a positive correlation between MMSE score and plasma soluble fractalkine level in the patients with AD. In addition, it has been demonstrated that the expression of the gene coding for CX3CL1 was increased in the brain of patients with AD compared to controls, mostly in early stages of AD [70]. Moreover, authors studied the association between CX3CR1 genetic variants in a Spanish cohort, and found that the CX3CR-1-V249I variant was positively correlated with neurofibrillary pathology in particular with late onset AD patients [71]. All of these results combined lead us to consider CX3CL1 as a potential biomarker specially to detect early stages of AD.

### CXCL10

CXCL10 or IP-10 (interferon-gamma induced Protein) through its receptor CXCR3 is a chemotactic cytokine which has several biological effects [41]. CXCL10 is constitutively expressed in a subpopulation of astrocytes [72], while its expression can be induced by a wide number of cell types under inflammation such as lymphocytes, endothelial cells, monocytes or polynuclear neutrophils [73]. In our experimental conditions, we found that CXCL10 expression was decreased in PBMCs and U87i cells in the BBB model with PBMCs from patients with moderate AD compared to patients with mild AD. Interestingly, other authors showed that CXCL10 expression was increased in the CSF of patients with moderate AD and decreased in patients with severe AD [74, 75]. On the contrary, in another study no difference has been found between patients with AD and control patients in CSF and serum levels of CXCL10 [43]. CXCL10 seems to be implicated in the pathogenesis of AD, indeed authors showed that a deficiency in CXCR3 helped to prevent inflammation, and a lower amyloid burden and less cognitive dysfunctions have been observed in a mouse model of AD [76]. CXCL10 may also play a role in neuronal apoptosis [77], and seems to be implicated into neuronal death [41]. All those results lead us to consider CXCL10 as a rather negative chemotactic cytokine, and should be a good candidate as therapeutic target. Indeed, it could be interesting to slow down its expression especially in early stages, to modify the progression of AD.

It is interesting to note that most of recent trials using anti-inflammatory drugs have failed to prevent or delay the development of AD [78]. Some possible explanation could be that these treatments are given too lately in the pathogenesis of AD for them to be efficient. Indeed, Cuello *et al* [9] described that early inflammation may begin as soon as amyloid pathology (neurotoxic Aβ oligomeric) cross a threshold, even before the formation of amyloid plaques. While a secondary process including late inflammation may appear in response to amyloid plaques. Anti-inflammatory drugs should be given in MCI patients or even with patients with no cognitive dysfunction in order to be effective. Another answer would be that these treatments are trying to stop inflammation as a “whole” while some signals could still be useful to delay the progression of AD, that is why targeted therapy could be an alternative and more successful solution. Some authors are already trying to assess how targeting chemokines could be useful in multiple pathologies [79].

## Conclusion

This study highlights the usefulness of a human blood brain barrier model to detect some changes of chemokine expression during the progression of AD just by using PBMCs from patients and their impact in BBB model. Indeed, the five tested chemokines have shown to be expressed differently regarding the severity of AD. We observed a decrease in the expression of CCL2 in PBMCs, hCMEC/D3 cells and U87i cells in the BBB model with PBMCs from moderate AD patients compared to mild AD patients, which could be used as a potential biomarker to discriminate later stages of AD. An early increase in the CX3CL1 production was also shown in both M1 and M2 culture media in the BBB model with PBMCs from mild AD patients. This early increase of CX3CL1 could potentially help to discriminate early stages of AD. We also found a decrease in both CCL5 and CXCL10 levels. Indeed, CCL5 expression was decreased in hCMEC/D3, U87i, and U87w cells and in the culture media M2 in the BBB model with PBMCs obtained from moderate AD patients compared to mild AD patients. Concerning CXCL10, its production was decreased in PBMCs and U87i cells in the BBB model with PBMCs from moderate AD patients compared to mild AD patients. Although these results come from an *in vitro* BBB model using hCMEC / D3 endothelial cell line known to have some limits compared to primary cultures, these variations in the chemokine expression could provide useful biomarkers to follow-up the progression of AD and for potential therapeutic targets.

## Supporting information

S1 Fig Permeability assay values for Fluorescein Isothiocyanate-Dextran (FD4) in the human BBB model after one hour of incubation under two conditions as described in “methods” section: inserts without cells (Control) and BBB model without PBMCs. The endothelial permeability coefficients (Pe) are expressed in cm/s and bars represented mean ± SEM of 10 independent experiments. The mean values of BBB permeability for FD4 were: 4.50 ± 0.62 (x10^-6^ cm/s) in the BBB model without PBMCs compared to 32.50 ± 2.56 (x10^-6^ cm/s) in control. Fluorescence (λex = 485 nm and λem = 515 nm) was measured by using a Varioskan Flash^®^ microplate reader (Fisher ThermoScientific). ***P < 0.005 compared to control by a Mann-Whitney’s test.
(PDF)

S2 Fig Co-Immunolabelling of nuclei with different cell type marker in U87 cell line. (A) Co-immunostaining of nuclei (DAPI, blue channel) with GFAP marker (green channel) in U87 cells. Scale bars: 50 μM (B) Co-immunostaining of nuclei (DAPI, blue channel) with NSE marker (red channel) in U87 cells. Scale bars: 25 μM (C) Co-immunostaining of nuclei (DAPI, blue channel) with IBA1 marker (red channel) in U87 cells. Scale bars: 50 μm.
(PDF)

## Abbreviations

Aβ: β-amyloid peptide
AD: Alzheimer’s disease
BBB: blood brain barrier
bFGF: human basic Fibroblast Growth Factor
CCL: CC chemokine ligand
CNS: central nervous system
CSF: cerebrospinal fluid
CX3CR1: C-X3-C Motif Chemokine Receptor 1
DAPI, 4': 6-diamidino-2-phenylindole
EBM-2: Endothelial Basal Medium-2
FD4: fluorescein isothiocyanate-dextran 4kDa
HBSS: Hank's Balanced Salt Solution
hCMEC/D3 or D3: human endothelial cell line from the immortalized cerebral microvascular endothelial cell line
GFAP: glial fibrillary acidic protein
Iba1: ionized calcium-binding adapter molecule 1
IP-10: interferon-gamma induced Protein-10
LPS: lipopolysaccharide
MCI: mild cognitive impairment
MCP-1: Monocyte Chemoattractant Proteins 1
MFIs: median fluorescence intensities
MIP1-β: Macrophage Inflammatory Protein-1-β
MMSE: Mini Mental State Examination
NaF: sodium fluoride
NINCDS-ADRDA: National Institute of Neurological and Communicative Disorders and the Stroke-Alzheimer’s Disease and Related Disorders Association
NSE: neuronal specific enolase
PBMCs/PB: peripheral blood mononuclear cells
PFA: paraformaldehyde
PMSF: phenylmethylsulfonyl fluoride
PHA: phytohaemagglutin
PS: penicillin/streptomycin
RANTES: Regulated on Activation, Normal T Expressed and Secreted
TEER: Trans-endothelial electric resistance
