# Supplementary material for: Differential chemokine expression under the control of peripheral blood mononuclear cells issued from Alzheimer’s patients in a human blood brain barrier model
Source: PLoS One. 2018 Aug 9;13(8):e0201232. doi: 10.1371/journal.pone.0201232 (PMC6084889; doi:10.1371/journal.pone.0201232)
Supplement: S1 Fig — The endothelial permeability coefficients (Pe) are expressed in cm/s and bars represented mean ± SEM of 10 independent experiments. The mean values of BBB permeability for FD4 were: 4.50 ± 0.62 (x10-6 cm/s) in the BBB model without PBMCs compared to 32.50 ± 2.56 (x10-6 cm/s) in control. Fluorescence (λex = 485 nm and λem = 515 nm) was measured by using a Varioskan Flash® microplate reader (Fisher ThermoScientific). ***P < 0.005 compared to control by a Mann-Whitney’s test. (PDF) [file pone.0201232.s001.pdf]

Supporting information 1 VERITE et al

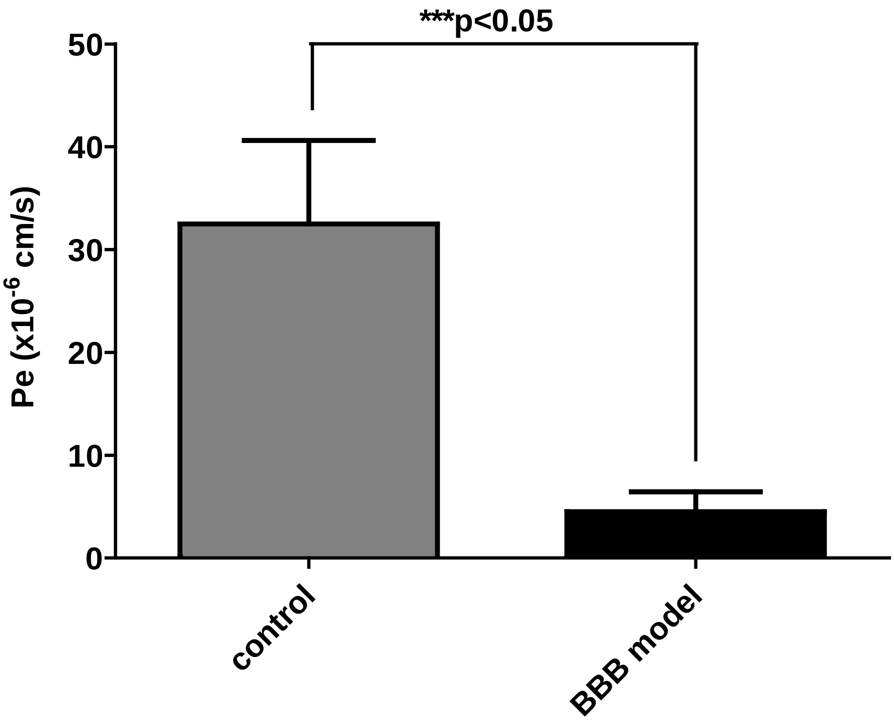

Supporting information 1

Permeability assay values for Fluorescein Isothiocyanate-Dextran (FD4) in the human BBB model after one hour of incubation under two conditions as described in "methods" section: inserts without cells (Control) and BBB model without PBMCs. The endothelial permeability coefficients (Pe) are expressed in cm/s and bars represented mean ± SEM of 10 independent experiments. The mean values of BBB permeability for FD4 were: 4.50 ± 0.62 (x10^{-6} cm/s) in the BBB model without PBMCs compared to 32.50 ± 2.56 (x10^{-6} cm/s) in control. Fluorescence (λex = 485 nm and λem = 515 nm) was measured by using a Varioskan Flash® microplate reader (Fisher ThermoScientific). ***P < 0.005 compared to control by a Mann-Whitney's test.
