# Supplementary material for: Differential chemokine expression under the control of peripheral blood mononuclear cells issued from Alzheimer’s patients in a human blood brain barrier model
Source: PLoS One. 2018 Aug 9;13(8):e0201232. doi: 10.1371/journal.pone.0201232 (PMC6084889; doi:10.1371/journal.pone.0201232)
Supplement: S2 Fig — (A) Co-immunostaining of nuclei (DAPI, blue channel) with GFAP marker (green channel) in U87 cells. Scale bars: 50 μM (B) Co-immunostaining of nuclei (DAPI, blue channel) with NSE marker (red channel) in U87 cells. Scale bars: 25 μM (C) Co-immunostaining of nuclei (DAPI, blue channel) with IBA1 marker (red channel) in U87 cells. Scale bars: 50 μm. (PDF) [file pone.0201232.s002.pdf]

Supporting information 2 VERITE et al

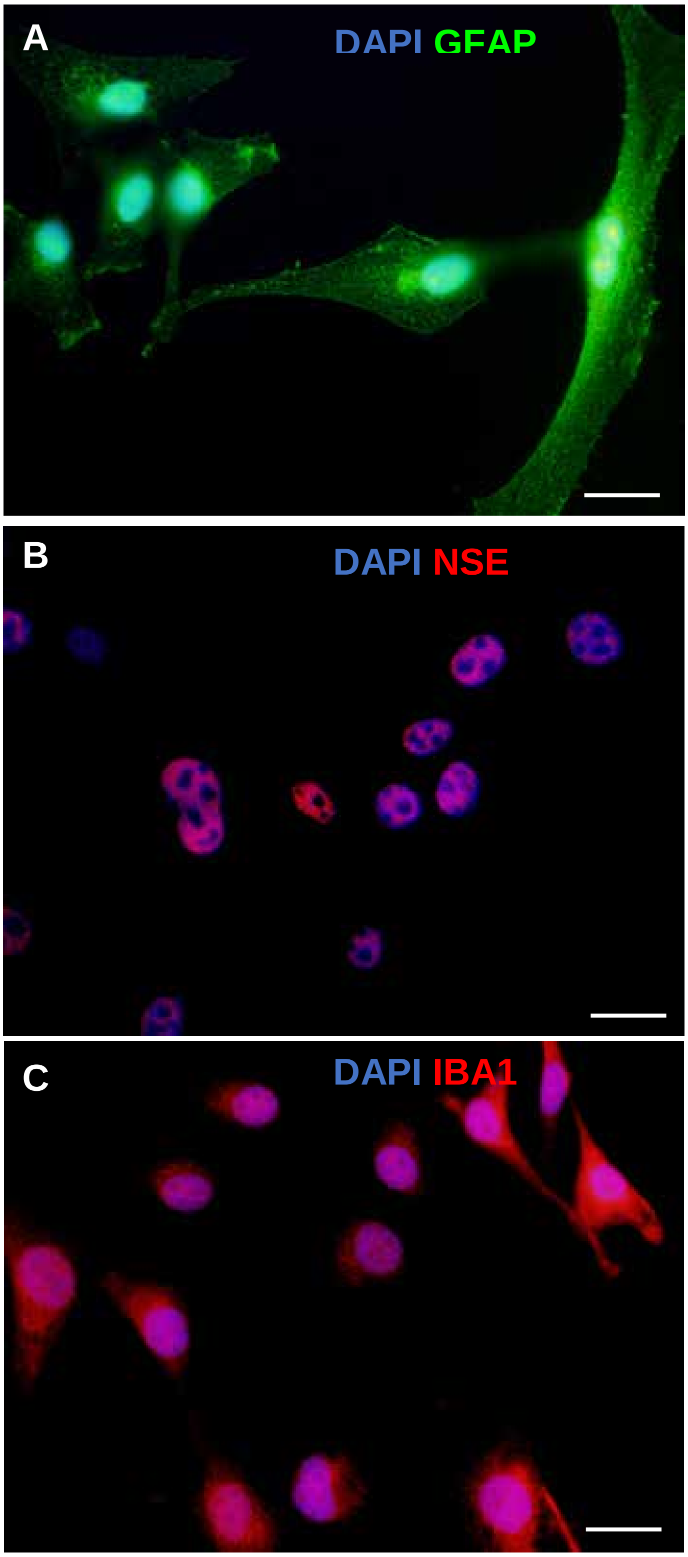

Supporting information 2:

Co-Immunolabelling of nuclei with different cell type marker in U87 cell line. (A) Co-immunostaining of nuclei (DAPI, blue channel) with GFAP marker (green channel) in U87 cells. Scale bars: 50 µM (B) Co-immunostaining of nuclei (DAPI, blue channel) with NSE marker (red channel) in U87 cells. Scale bars: 25 µM (C) Co-immunostaining of nuclei (DAPI, blue channel) with IBA1 marker (red channel) in U87 cells. Scale bars: 50 µm.
